# Long-term clinical efficacy of drug-coated balloon angioplasty for TASCII C/D femoropopliteal lesions in older patients with chronic limb-threatening ischemia: A retrospective study

**DOI:** 10.1097/MD.0000000000039331

**Published:** 2024-08-16

**Authors:** Feng Zhang, Hai-Xia Song, Li-Hua Zheng, Yan-Bo An, Peng Liu

**Affiliations:** a Department of Vascular and Endovascular Surgery, Hebei Key Laboratory of Colorectal Cancer Precision Diagnosis and Treatment, The First Hospital of Hebei Medical University, Shijiazhuang, Hebei, PR China; b Department of Neurology, Shijiazhuang People’s Hospital, Shijiazhuang, Hebei, PR China.

**Keywords:** chronic limb-threatening ischemia, drug-coated balloon, femoropopliteal TASCII C/D lesions, restenosis, target lesion revascularization

## Abstract

This study aimed to evaluate the long-term clinical outcomes of drug-coated drug (DCB) angioplasty for long femoropopliteal lesions in older patients with chronic limb-threatening ischemia (CLTI). In this multi-center retrospective study, we enrolled 119 patients with CLTI due to Trans-Atlantic Inter-Society Consensus (TASCII) C/D femoropopliteal lesions who underwent DCB angioplasty. A total of 119 patients with 122 limbs (TASCII C = 67, 54.9%; TASCII D = 55, 45.1%) were enrolled. At 36-month follow-up, primary patency, assisted primary patency, secondary patency, and freedom from target lesion revascularization were 47.3%, 49.8%, 59.5%, and 62.7%, respectively, and there was a significant improvement over baseline in Rutherford class (*P* < .001) and ankle-brachial index measurements (*P* < .001). Complex target lesions (*P* = .017) and 1 stenosis-free outflow vessel (*P* = .001) were risk predictors of freedom from clinically driven target lesion revascularization. Complex target lesions (*P* = .044), diabetes (*P* = .007), and 1 stenosis-free outflow vessel (*P* = .003) were risk predictors of restenosis. At 2 months, the ulcer healing rate was 96.3% (26/27). At 36 months, the limb salvage and survival rates were 85.8% and 83.3%, respectively. DCB angioplasty were safe and effective for older patients with CLTI attributable to femoropopliteal TASCII C/D lesions.

## 1. Introduction

Chronic limb-threatening ischemia (CLTI) is an end-stage of symptomatic peripheral arterial disease (PAD), with a higher incidence in older people.^[1–3]^ Endovascular therapy (EVT) is recommended for femoropopliteal lesions shorter than 25 cm.^[4,5]^ Currently, open surgery as the gold standard to treat Trans-Atlantic Inter-Society Consensus (TASC) II D continues to be debated.^[4,6]^ However, patients presenting with CLTI are usually elderly and have poor physical fitness, putting them at high risk for complications with open surgery. In recent years, with advances in endovascular techniques and medical devices, EVT, especially with drug-coated balloon (DCB) and/or drug-eluting stents (DES), has achieved good clinical benefits in shorter femoropopliteal lesions,^[7–9]^ but the effectiveness of DCB angioplasty is often challenged by long lesion length and the presence of chronic total occlusion, and data from such studies were less informative in assessing the efficacy of EVT in TASCII C/D femoropopliteal lesions, especially in Asian patients.^[10,11]^ Therefore, we aimed to evaluate the safety and efficacy of DCB angioplasty for the treatment of TASCII C/D femoropopliteal lesions in older patients with CLTI over 3 years of follow-up.

## 2. Materials and methods

### 2.1. Patients

Patients who underwent DCB angioplasty for lower-limb TASCII C/D femoropopliteal lesions with CLTI at the First Hospital of Hebei Medical University and Shijiazhuang People’s Hospital between November 2019 and December 2021 were recruited for this study. The flow diagram of this study is presented in Figure S1, Supplemental Digital Content, http://links.lww.com/MD/N375. All enrolled patients underwent ankle-brachial index (ABI), ultrasound Doppler imaging, and computed tomographic angiography. The inclusion and exclusion criteria for the study are listed in Table S1, Supplemental Digital Content, http://links.lww.com/MD/N376. This study was conducted in accordance with the principles of the Declaration of Helsinki and followed the Reporting of Observational Studies in Epidemiology guidelines. The study protocol was reviewed and approved by the Medical Ethics Committee (No. 2020345).

### 2.2. Treatment

All procedures were performed according to the standards of femoropopliteal artery endovascular revascularization via either a contralateral or an ipsilateral approach. After sheath placement, an intravenous bolus of 100 UI/kg unfractionated heparin was administered, and the activated coagulation time was maintained at >200 s. The target vessel was first opened with intraluminal recanalization unless the subintimal technique was required. Lesion predilatation was performed before drug-coated angioplasty (Lutonix 035; Becton, Dickinson and Company, Franklin Lakes, NJ). In cases of severe flow-limiting dissection or suboptimal angiographic results with significant recoil and/or residual stenosis, a bail-out bare metal stent (BMS) was implanted. The stent dimensions were chosen by visual estimation to fit the vessel diameter perfectly, with a length exceeding the lesion length by 5 to 10 mm proximally and distally. The same type of nitinol self-expanding stent (Protege Everflex ev3, Plymouth, MN) and cover stent (GORE VIABAHN Endoprosthesis, W. L. Gore & Associates, Flagstaff, AZ) were implanted. Closure of the artery was accomplished using an arterial closure device (Starclose, Abbott Vascular, Green Oaks, IL).

### 2.3. Drug administration and follow-up

Preoperative dual antiplatelet therapy (aspirin 100 mg/day and clopidogrel 75 mg/day) was administered for at least 3 days. Postoperatively, the patients were required to receive aspirin (100 mg/day) and clopidogrel (75 mg/day) for 6 months, followed by aspirin or clopidogrel alone. Follow-up visits, including ABI, clinical examination, and ultrasound Doppler, were scheduled 30 days and 6 months after the index procedure and every 6 months thereafter. Visits were conducted via a combination of outpatient and telephone reviews.

### 2.4. Definitions

These definitions are presented in Table S2, Supplemental Digital Content, http://links.lww.com/MD/N377. Rutherford grade 4 was defined as rest pain, Rutherford grade 5 was defined as ulceration, and Rutherford grade 6 was defined as gangrene. Calcification was classified as mild, moderate, or severe.^[12]^ Recurrent symptoms were defined as Rutherford class 3 to 6 on follow up. The size of the ischemic ulcers was calculated using the area method. Patency rate was defined as 1 minus the patency endpoint cumulative incidence. Freedom from Clinically driven target lesion revascularization (CD-TLR) was defined as the 1 minus CD-TLR endpoint cumulative incidence.

### 2.5. Statistical methods

Data were analyzed using SPSS 26.0, and Stata 15.1. Continuous variables were summarized as mean ± standard deviation or median. Categorical variables are summarized as counts and percentages. All data were tested for normality using the Kolmogorov–Smirnov test. Deaths were competing risk events for limb-related outcomes. Cumulative incidence was generated using competing risk analyses. A competing risk regression model was used to analyze the association between various covariates and restenosis and CD-TLR. Statistical significance was set at *P* < .05.

## 3. Results

### 3.1. Patient characteristics

The clinical data of 119 patients with 122 lower limbs were enrolled. Patient characteristics are shown in Table 1. Fifty-nine patients (49.6%) were with ≥2 risk features. The baseline lesion characteristics and procedural outcomes are shown in Table 2. The mean follow-up duration was 36 (24, 40) months.

**Table 1:** Patient characteristics.

Characteristics	n (%)
Age, years	68.5 (61, 75)
Male sex	85 (71.4)
Smoking (never/former/current)*	37 (31.1)/21 (17.6)/61 (51.3)
BMI＞24*	56 (47.1)
Coronary artery disease	23 (19.3)
Cerebrovascular disease	12 (10.1)
Hypertension*	92 (77.3)
Dyslipidaemia*	62 (52.1)
Hyperhomocystinemia*	9 (7.6)
Type 2 diabetes*	15 (12.3)
Chronic kidney disease*	18 (15.1)
Follow-up time, months	36 (24, 40)

For age and follow up time indicated “median (IQR).” For risk features indicated “*.”

BMI = Body mass index.

**Table 2:** Baseline lesion characteristics and procedure outcomes.

Characteristicsss	n (%)
TASCII C/D	67 (54.9)/55 (45.1)
Limb	
Left	64 (52.5)
Right	58 (47.5)
Rutherford class 4/5/6	76 (62.3)/27 (22.1)/19 (15.6)
Type of anesthesia	
Local	117 (95.9)
General	5 (4.1)
Access site	
Crossover	85 (69.7)
Anterograde	37 (30.3)
Chronic total occlusion	95 (77.9)
Target lesion length, mm	227 (59)
Reference vessel diameter, mm	5 (4, 5)
Complex lesions	17 (13.9)
Calcification	
None/mild	75 (61.5)
Moderate	32 (25.2)
Severe	15 (12.3)
Number of run-off outflow	
1	80 (65.6)
2	33 (27.0)
3	9 (7.4)
Type of EVT	
DCB	73 (59.8)
DCB + bail out stenting	49 (40.2)
Diameter of stent	
6 mm	61 (76.3)
5 mm	19 (23.7)
Length of hospital stay, days	13 (9, 17)
Operating time, minutes	93.1 (32.7)
Contrast dosage, mL	93.0 (31.2)

For length of hospital stay and reference vessel diameter indicated “median (IQR).” For Target lesion length, operating time and contrast dosage indicated “mean (SD).”

DCB = drug-coated balloon, EVT = endovascular therapy, PTA = percutaneous transluminal angioplasty, TASCII = Trans-Atlantic Inter-Society Consensus-II.

### 3.2. Clinical improvement

At 1, 12, 24, and 36 months, the mean Rutherford classification decreased from 4.53 ± 0.75 (n = 122) at baseline to 0.77 ± 0.67 (n = 120), 0.81 ± 0.69 (n = 118), 0.86 ± 0.79 (n = 107), and 0.93 ± 0.88 (n = 104), respectively. The mean ABI increased from 0.19 ± 0.11 (n = 122) before the procedure to 0.89 ± 0.08 (n = 116), 0.82 ± 0.09 (n = 113), 0.78 ± 0.07 (n = 100), and 0.73 ± 0.09 (n = 97), respectively. The ulcer size range was 1.3 to 5.2 cm^2^, with an average of 3.3 ± 1.0 cm^2^. At 1 and 2 months, the ulcer healing rates were 88.9% (24/27) and 96.3% (26/27), respectively. The ulcer healing time was 19.3 ± 11.9 days.

### 3.3. Vessel patency

**Figure 1.**
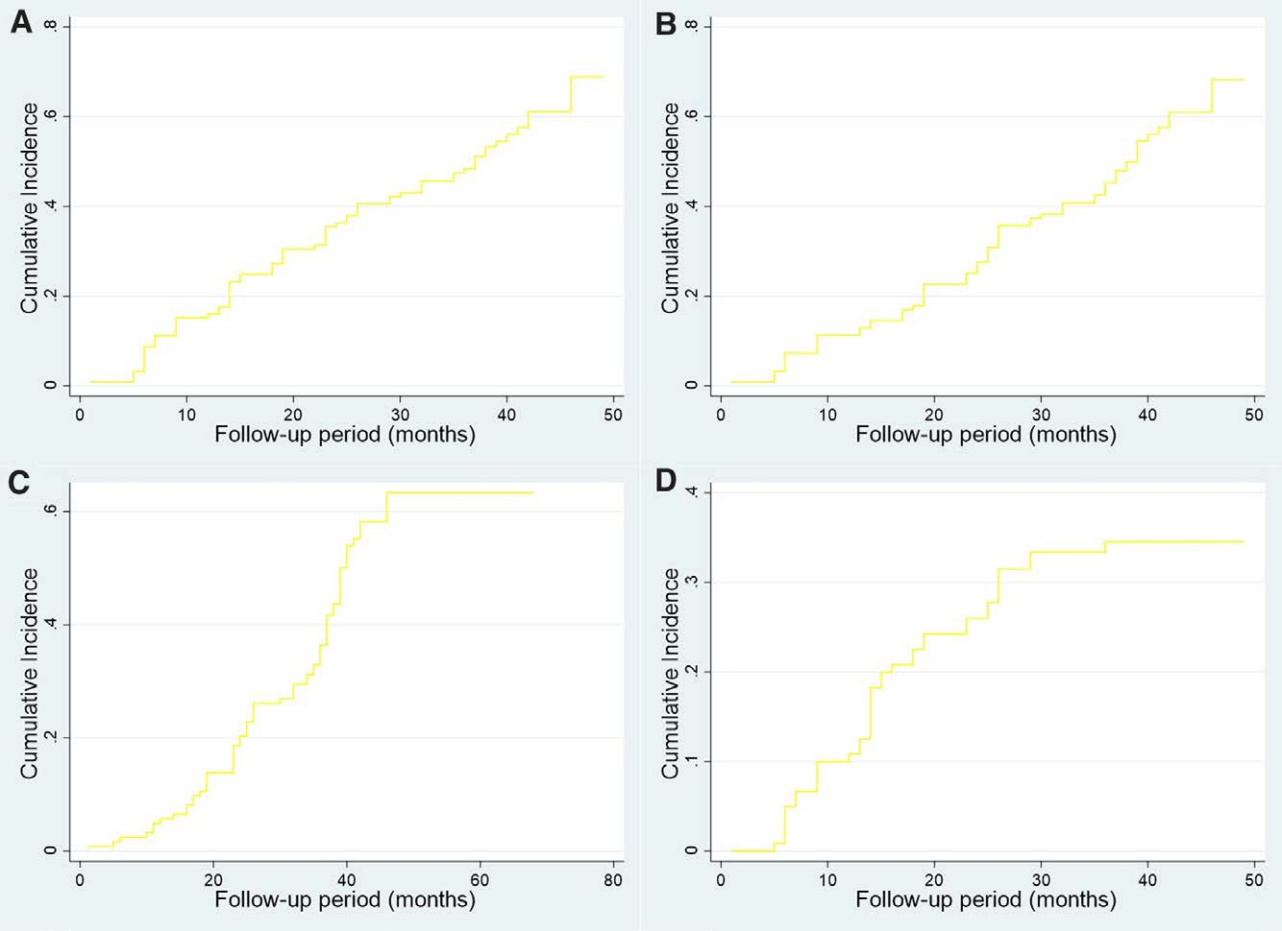
Competing-risk regression shows cumulative incidence over follow-up period. (A) Primary patency endpoint. (B) Assisted-primary patency endpoint. (C) Secondary patency endpoint. (D) TLR endpoint.

At 12, 24, and 36 months, the primary patency rates were 83.2%, 60.1%, and 47.3%, respectively; assisted primary patency rates were 88.2%, 70.2%, and 49.8%, respectively; secondary patency rates were 94.1%, 77.9%, and 59.5%, respectively; freedom from CD-TLR rates were 88.9%, 72.7%, and 62.7%, respectively. Competing risk regression shows the cumulative incidence of endpoints over the period in Figure 1. Competing risk analysis showed that complex target lesions (*P* = .017) and 1 stenosis-free outflow vessel (*P* = .001) were risk predictors of CD-TLR (Table S3, Supplemental Digital Content, http://links.lww.com/MD/N378).

### 3.4. Periprocedural complications

During the perioperative period, a total of 9 procedure-related complications occurred: coronary heart disease (n = 3), embolization (n = 2), thrombosis in-stent (n = 1), ischemic stroke (n = 1), pseudoaneurysm (n = 1), retroperitoneal hematoma (n = 1), and acute compartment (n = 1). Among them, 2 cases died, and the rest were discharged after conservative or surgical treatment.

### 3.5. Restenosis

At the time of follow-up, 63.9% (78/122) of the limbs developed restenosis and 51.73% (40/78) were symptomatic. Competing risk analysis showed that complex target lesions (*P* = .044), diabetes (*P* = .007), and 1 stenosis-free outflow vessel (*P* = .003) were risk predictors of restenosis (Table S4, Supplemental Digital Content, http://links.lww.com/MD/N379). Thirty-nine symptomatic patients underwent EVT including covered stents (n = 12), DCBs (n = 21), mechanical thrombectomy (n = 9), and catheter-directed thrombolysis (n = 2).

### 3.6. Amputation

At 12, 24, and 36 months, amputation-free survival were 88.3%, 85.7%, and 83.3%, respectively. Nineteen amputations were performed, including 7 major amputations and 12 minor amputations. The incision achieved primary healing with no complications in 17 patients (17/19, 89.5%).

### 3.7. Survival rate

At 12, 24, and 36 months, survival rates were 96.6%, 87.8%, and 85.8%, respectively. Sixteen deaths occurred due to cardiovascular disease (including 2 with periprocedural myocardial infarction) (n = 6), cancer (n = 2), multiorgan dysfunction (n = 1), intracerebral hemorrhage (n = 1), traffic accident (n = 1), COVID-19 with respiratory failure (n = 4), and natural causes (n = 1).

## 4. Discussion

In recent years, EVT has become a basic option for managing patients with CLTI.^[13]^ Advances in endovascular techniques and products, especially DCB and DES for EVT, have improved the efficacy of EVT for longer femoropopliteal lesions.^[14,15]^ Our multicenter, observational study documents the 3-year clinical outcomes of DCB angioplasty of TASCII C/D femoropopliteal lesions with CLTI. It included patients with both long femoropopliteal lesions and chronic total occlusions, which have traditionally not been included in previous studies.

In a real-world study among Asian populations, researchers found that DCB was safe and effective when used for long femoropopliteal lesions, and the primary patency rate at 12 months was 82.1% in a multicenter cohort^[16]^ and 78.8% in a single-arm trial,^[17]^ consistent with our report. In our study, the 24-month primary patency rate was 55.1% and the freedom from CD-TLR rate was 72.7%, consistent with previous report with rates of 50.0% and 72.7%,^[18]^ but lesions in our study were longer than those mentioned above. A prospective, multicenter, single-arm IN.PACT Global Study evaluating the performance of the IN.PACT Admiral DCB in real-world patients with femoropopliteal occlusion of mean lesion length (12.1 ± 9.5 cm) reported that the Kaplan–Meier estimated of freedom from CD-TLR with CLTI through 36 months was 67.6%.^[19]^ At 12 months, the clinical success rate was 98.4% in this study, which was higher than 87.2% and 72.7% in other reports.^[17,20]^ The better clinical outcomes and high patency in long and chronic total occlusion may be due to the following reasons. First, more stringent inclusion and exclusion criteria were developed for this study. Second, DCB angioplasty is an effective alternative to BMS and/or plain old balloon angioplasty, with promising patency. Third, operators in our center will try their best to use the intraluminal method other than the subintimal method and prefer to predilate to have larger luminal diameters to decrease the risk of dissection and residual stenosis. Finally, the same brand name stent used to treat the target lesion may be a significant factor in achieving a higher patency rate. Furthermore, treating femoropopliteal lesions using The BioMimics 3D stent resulted in good 3-year outcomes with freedom from CD-TLR significantly higher (78.0%) compared to 54.4% in our study, but lesions in our trial were approximately twice as long as those in MIMICS 3D (lesion length of 125.9 ± 91.0 mm).^[21]^ The IN.PACT Global DCB registry had an approximately half as long as lesion length (120.9 mm) compared to our study, and reported 76.9% freedom from TLR at 3 years.^[19]^ We also found that the 24-month primary patency rate and freedom from CD-TLR were higher than our previous literature with rates of 34.7% and 67.0%, respectively,^[22]^ because the length of the target lesions (227 ± 59 mm) was shorter than that in previous study (286 ± 42 mm) that included only femoropopliteal TASCII D lesions.

Restenosis rates after primary stent implantation were as high as 30% to 50% during the follow-up period.^[23]^ The restenosis rates in this study were 30.9% and 52.7% at 24 months and 36 months, respectively. The failure rates were higher with low-dose DCB, severe calcification, chronic total occlusion, and longer target lesions.^[8,24]^ Bailout BMS remains a routine treatment for the management of femoropopliteal artery lesions in this study, although the patency benefit of DES has been demonstrated.^[25,26]^ EVT was performed in 51.3% (40/78) of patients with recurrent symptoms, in accordance with a previous report.^[27]^ Both sirolimus and paclitaxel have been shown to be effective in limiting restenosis following EVT. Zeller et al found that a new type of sustained-limus-release DCB appeared to effectively and safely inhibit restenosis, improving outcomes in patients with femoropopliteal lesions over 6 months.^[28]^ Furthermore, the SurVeil DCB was considered to further optimize efficacy while simultaneously providing lower drug loading on the balloon and lower systemic exposure of paclitaxel.^[29]^ However, clinical outcomes should be compared to other DCBs.

Hybrid therapy is a potential solution for the high restenosis rate of postoperative involvement of femoral artery bifurcation lesions is always being discussed. Recently, EVT has been proposed as a potential alternative because of its low invasiveness and short hospitalization duration. However, based on the evidence available, thromboendarterectomy may still be considered the standard treatment.^[30,31]^ With adherence to existing specific guidelines, we consider that the combination therapy of thromboendarterectomy of the common femoral artery and EVT for the femoropopliteal artery may be a suitable hybrid therapy for TASC II C/D femoropopliteal lesions involving the femoral artery bifurcation.

The mortality rate in this study was high for the following reasons: first, the patients included in the study were elderly. Second, with diagnosed large-vessel PAD, life expectancy is shorter than without diagnosis,^[32]^ and extensive atherosclerosis of crural vessels is associated with long-term cardiovascular mortality in patients with symptomatic PAD.^[33]^ Third, the spread of coronavirus disease 2019 in recent years has resulted in a significant increase in mortality among older individuals.

Hyperglycemia triggers endothelial dysfunction and subsequent neointimal and vascular smooth muscle cell proliferation, which is an established mechanism underlying restenosis. Our study suggests that diabetes is associated with poor patency of target lesions. Pasqual Mone et al also found that hyperglycemia drives stent restenosis in ST-elevation myocardial infarction patients independent of diabetes.^[34]^ The individualized prediction nomogram incorporating hyperglycemia can be used to facilitate early identification of patients undergoing percutaneous coronary intervention at higher risk of in-stent restenosis in Asian population.^[35]^ A systematic overview summarizing the clinical presentation of restenosis concluded that the diabetes is a strong determinant of neointimal hyperplasia, a statistically significant predictor of restenosis.^[36]^ Besides, the previous animal study demonstrated that the protective effects are maintained in severe hyperglycemia, and treatment with glucagon-like peptide-1 receptor agonists represents potentially effective pharmacological therapy following angioplasty in patients with diabetes.^[37]^ Indeed, we should emphasize the importance of glycemic control in diabetic patients who underwent revascularization to decrease restenosis in effective secondary prevention.

### 4.1. Limitations

This study had several limitations. First, it was a multi-center, retrospective arm study of DCB angioplasty, with a lack of head-to-head comparison with vein bypass. Second, with inclusion eligibility confined to Rutherford grades 4 to 6, the study sample was representative of patients with CLTI but not of those with intermittent claudication. Finally, owing to the small sample size, our study is likely to be underpowered and inconclusive. Larger studies with a prospective design and longer follow-up are advisable to clearly establish the clinical impact of DCB angioplasty.

## 5. Conclusion

In this study, DCB angioplasty demonstrated 36-month primary patency and freedom from CD-TLR and achieved clinical outcomes in long and occlusive femoropopliteal lesions with symptomatic improvement, wound healing, and functional limb preservation. DCB angioplasty for the treatment of femoropopliteal TASCII C/D lesions in older patients with CLTI were safe and effective.

## Author contributions

**Conceptualization:** Peng Liu.

**Formal analysis:** Hai-Xia Song.

**Investigation:** Hai-Xia Song.

**Methodology:** Yan-Bo An.

**Software:** Yan-Bo An.

**Validation:** Li-Hua Zheng.

**Visualization:** Li-Hua Zheng.

**Writing – original draft:** Peng Liu.

**Writing – review & editing:** Feng Zhang.

## Supplementary Material


