# Supplementary material for: Long-term clinical efficacy of drug-coated balloon angioplasty for TASCII C/D femoropopliteal lesions in older patients with chronic limb-threatening ischemia: A retrospective study
Source: Medicine (Baltimore). 2024 Aug 16;103(33):e39331. doi: 10.1097/MD.0000000000039331 (PMC11332706; doi:10.1097/MD.0000000000039331)
Supplement: Supplementary file 1 [file medi-103-e39331-s001.docx]

Diagnosed PAD with CLTI due to femoropopliteal TASCII C/D target lesions (187 patients, 190 limbs)

Enrollment after technical success

(119 patients, 122 limbs)

The guidewire did not cross the target lesion (2 patients, 2 limbs)

Endovascular therapy

(121 patients, 124 limbs)

Bypass surgery (37 patients, 37 limb)

Acceptance of initial surgery

(158 patients with 161 limbs)

Unsuitable for surgery (10 patients, 10 limbs)

Refused treatment (5 patients, 5 limbs)

Previous endovascular therapy (11 patients, 11 limbs)

Life expectancy＜2 years (3 patients, 3 limbs)

Supplementary Figure 1. Participant flow diagram of all patients with CLTI due to femoropopliteal TASCII C/D lesions who had presented to our hospital between November 2019 to December 2021.
