# Supplementary material for: Long-term clinical efficacy of drug-coated balloon angioplasty for TASCII C/D femoropopliteal lesions in older patients with chronic limb-threatening ischemia: A retrospective study
Source: Medicine (Baltimore). 2024 Aug 16;103(33):e39331. doi: 10.1097/MD.0000000000039331 (PMC11332706; doi:10.1097/MD.0000000000039331)
Supplement: Supplementary file 2 [file medi-103-e39331-s002.docx]

| Supplementary Table 1. Inclusion and exclusion Criteria | |
| --- | --- |
| Inclusion criteria | Exclusion criteria |
| 1. Age ＞60 years 2. Diagnosis of CTLI according to Rutherford stages 4, 5 and 6^2^ 3. TASCII C/D femoropopliteal lesions according to the TASCII guideline^1^ 4. At least one infragenicular artery (stenosis ＜30%) extending to the ankle and adequate femoropopliteal outflow defined as patency of the iliac artery (stenosis ＜30%) 5. All patients or their legal representatives who provided written informed consent | 1. Known allergies to anticoagulant/antiplatelet therapy 2. Contraindications of antiplatelet and anticoagulant therapy 3. Acute ischemia or arterial thrombosis in the target vessel 4. History of revascularization 5. Complicated with vasculitis 6. Clinical syndromes of CLTI caused by venous, traumatic, embolic, and nonatherosclerotic etiologies 7. Life expectancy ＜ 2 years |
| CLTI, Chronic Limb-Threatening Ischemia; TASCII, Trans-Atlantic Inter-Society Consensus-II. | |
