# Supplementary material for: Long-term clinical efficacy of drug-coated balloon angioplasty for TASCII C/D femoropopliteal lesions in older patients with chronic limb-threatening ischemia: A retrospective study
Source: Medicine (Baltimore). 2024 Aug 16;103(33):e39331. doi: 10.1097/MD.0000000000039331 (PMC11332706; doi:10.1097/MD.0000000000039331)
Supplement: Supplementary file 3 [file medi-103-e39331-s003.docx]

| Supplementary Table 2. Definitions | |
| --- | --- |
| Characteristics | Definitions |
| CLTI | PAD in combination with rest pain, gangrene, or a lower limb ulceration ＞2 weeks duration |
| TASCII C lesion | Multiple stenoses or occlusions totaling ＞15 cm with or without heavy calcification |
| TASCII D lesion | Chronic total occlusions of CFA or SFA ＞20 cm, involving the proximal popliteal artery lesions |
| Moderate calcification | Radiopaque density seen on one side of the artery |
| Severe calcification | Radiopaque density on both sides of the artery |
| Device success | Successful delivery, inflation, deflation, and retrieval of the device |
| Technical success | Final residual stenosis ≤ 30% and positive blood flow measured by the investigators on the procedural completion angiogram |
| Clinical success | Wound healing and rest pain resolution at 3 months |
| Primary patency | Freedom from restenosis without any other revascularization in the target lesions |
| Assisted-primary patency | Patency with a secondary EVT performed in stenotic segment |
| Secondary patency | Patency with a secondary EVT performed for reocclusion in a subsequently patent vessel |
| Restenosis | ＞ 50% stenosis by angiography and ＞2.4 of the peak systolic velocity ratio by duplex scan |
| Major amputation | Any procedure that results in amputation above the level of ankle |
| Minor amputation | Amputation at the level of the ankle or below |
| Complex lesions | Involving femoral trifurcation including the CFA, origin of the PFA, or flush occlusion of the SFA. |
| The length of the hospital stay | The number of days from the date of the index procedure through discharge or 30 days after the procedure, whichever came first |
| Perioperative death | Death within 30 days of the procedure |
| The indication of stent implantation | Final residual stenosis > 30% as measured visually or flow-limiting dissection |
| CD-TLR | Any reintervention due to a stenosis or occlusion in the previously treated lesion as well as the presence of recurrent symptoms |
| Ulcer healing time | From the surgery date to the time by which the ulcer had healed |
| CLTI, Chronic limb-threatening ischemia; PAD, Peripheral artery disease; TASCII, Trans-Atlantic Inter-Society Consensus-II; EVT, Endovascular therapy; CFA, Common femoral artery; SFA, Superficial femoral artery; PFA; Profound femoral artery; CD-TLR, Clinically driven target lesion revascularization. | |
