# Supplementary material for: Long-term clinical efficacy of drug-coated balloon angioplasty for TASCII C/D femoropopliteal lesions in older patients with chronic limb-threatening ischemia: A retrospective study
Source: Medicine (Baltimore). 2024 Aug 16;103(33):e39331. doi: 10.1097/MD.0000000000039331 (PMC11332706; doi:10.1097/MD.0000000000039331)
Supplement: Supplementary file 4 [file medi-103-e39331-s004.docx]

Supplementary Table 3. Competing risk analysis of factors associated with CD-TLR

| Variable | SHR | Std. Err. | z | P | [95% Conf. | Interval] |
| --- | --- | --- | --- | --- | --- | --- |
|  |  |  |  |  |  |  |
| TASCII D | 2.347866 | 1.752084 | 1.14 | 0.253 | .5438365 | 10.13627 |
| Calcification | .7574396 | .3183019 | -0.66 | 0.509 | .332387 | 1.726044 |
| hypertension | 1.282984 | .5638968 | 0.57 | 0.571 | .5421271 | 3.036275 |
| Gender | 1.191646 | .5197287 | 0.40 | 0.688 | .5068753 | 2.801517 |
| BMI | .7112238 | .3177389 | -0.76 | 0.446 | .2963005 | 1.707183 |
| Smoking | .6964425 | .3489143 | -0.72 | 0.470 | .260878 | 1.85923 |
| Dyslipidaemia | 1.921207 | .7304328 | 1.72 | 0.086 | .9119097 | 4.04759 |
| Diabetes | 2.051631 | 1.085647 | 1.36 | 0.174 | .727235 | 5.787936 |
| Chronic Kidney disease | .7849037 | .3848229 | -0.49 | 0.621 | .3002541 | 2.051841 |
| Chronic total occlusion | 1.013231 | .7432872 | 0.02 | 0.986 | .240593 | 4.267111 |
| Bail out stenting | 2.049377 | 1.099928 | 1.34 | 0.181 | .7157652 | 5.867768 |
| Run-off outflow | .2120918 | .0986331 | -3.33 | 0.001 | .0852459 | .5276847 |
| Complex target lesion | 2.625236 | 1.059221 | 2.39 | 0.017 | 1.190498 | 5.78906 |
|  |  |  |  |  |  |  |
|  |  |  |  |  |  |  |

CD-TLR, Clinically driven target lesion revascularization; TASCII, Trans-Atlantic Inter-Society Consensus-II; BMI, Body mass index.
