# Supplementary material for: Long-term clinical efficacy of drug-coated balloon angioplasty for TASCII C/D femoropopliteal lesions in older patients with chronic limb-threatening ischemia: A retrospective study
Source: Medicine (Baltimore). 2024 Aug 16;103(33):e39331. doi: 10.1097/MD.0000000000039331 (PMC11332706; doi:10.1097/MD.0000000000039331)
Supplement: Supplementary file 5 [file medi-103-e39331-s005.docx]

Supplementary Table 4. Competing risk analysis of factors associated with restenosis

| Variable | SHR | Std. Err. | z | P | [95% Conf. Interval] | |
| --- | --- | --- | --- | --- | --- | --- |
|  |  |  |  |  |  |  |
| TASCII D | 2.467934 | 1.754809 | 1.27 | 0.204 | .6124801 | 9.944317 |
| Calcification | .5487864 | .1935157 | -1.70 | 0.089 | .2749467 | 1.095363 |
| hypertension | .7754803 | .3400465 | -0.58 | 0.562 | .3283376 | 1.831559 |
| Gender | .7884733 | .2961685 | -0.63 | 0.527 | .3776212 | 1.646333 |
| BMI | .8356398 | .387114 | -0.39 | 0.698 | .3370513 | 2.071773 |
| Smoking | .5696617 | .2827228 | -1.13 | 0.257 | .2153605 | 1.506843 |
| Dyslipidaemia | 1.178945 | .4529533 | 0.43 | 0.668 | .5552132 | 2.503382 |
| Diabetes | 4.980558 | 2.938432 | 2.72 | 0.007 | 1.567061 | 15.82961 |
| Chronic Kidney disease | .5462453 | .2933869 | -1.13 | 0.260 | .1906381 | 1.565186 |
| Chronic total occlusion | .9269477 | .6727719 | -0.10 | 0.917 | .2234906 | 3.8446 |
| Bail out stenting | 3.360192 | 2.311086 | 1.76 | 0.078 | .872813 | 12.9362 |
| Run-off outflow | .2225663 | .1119891 | -2.99 | 0.003 | .0830154 | .5967055 |
| Complex target lesion | 2.498903 | 1.13761 | 2.01 | 0.044 | 1.02387 | 6.09893 |
|  |  |  |  |  |  |  |
|  |  |  |  |  |  |  |

TASCII, Trans-Atlantic Inter-Society Consensus-II; BMI, Body mass index.
